# Health Related Quality of Life in Juvenile-Onset Systemic Lupus Erythematosus: A Questionnaire-Based Study

**DOI:** 10.1007/s10995-023-03680-x

**Published:** 2023-06-12

**Authors:** Riham Eid, Ayman Hammad, Mai S. Korkor, Aya A. Fathy, Dena M. Abd El-Ghafaar, Shaimaa Rakha, Nashwa Hamdy

**Affiliations:** 1 Pediatric Nephrology Unit, Faculty of Medicine, Mansoura University Children’s Hospital, Mansoura University, Mansoura, 35561 Egypt; 2 Public Health and Community Department, Faculty of Medicine, Mansoura University, Mansoura, Egypt; 3 Rheumatology, Rehabilitation and Physical Medicine Department, Faculty of Medicine, Mansoura University, Mansoura, Egypt; 4 Pediatric Cardiology Unit, Faculty of Medicine, Mansoura University Children’s Hospital, Mansoura University, Mansoura, Egypt

**Keywords:** Children, Egypt, Lupus, Quality of life

## Abstract

**Objectives:**

This study aimed to evaluate health related quality of life (HRQOL) in Egyptian children with systemic lupus erythematosus (SLE) using 3 different tools.

**Methods:**

In this questionnaire-based study, 100 children with SLE were included. HRQOL was assessed using the Pediatric Quality of Life Inventory Generic Core Scales (PedsQL™ 4.0 GCS), PedsQL™ 3.0 Rheumatology Module (PedsQL3-RM) and the Simple Measure of the Impact of Lupus Erythematosus in Youngsters (SMILEY). SLE disease activity index (SLEDAI) was used to evaluate activity and SLE International Collaborating Clinics/ American College of Rheumatology Damage Index (SDI) was used to evaluate chronic damage.

**Results:**

All mean scores of PedsQL^TM^4.0 GCS domains in SLE patients were lower than published normative data and previously published results of Egyptian healthy controls (*p* < 0.001). All mean scores of PedsQL-3RM domains were significantly lower than published normative data except for the treatment and pain and hurt domains (*p* = 0.1, 0.2 respectively). SMILEY scores were low and the lowest domain scores was “Burden of SLE”. Longer duration of illness, higher cumulative steroid doses, higher SLEDAI and SDI scores and presence of obesity were associated with lower scores for all 3 tools (*p* < 0.001).

**Conclusion for Practice:**

The Arabic copies of PedsQL™ 4.0 GCS, PedsQL3-RM and SMILEY are easily used for Arabic speaking subjects and easily interpreted by physician and can be implemented for frequent monitoring of SLE HRQOL. Controlling the disease activity and using lowest doses of steroids and other immunosuppressive drugs are the corner stone strategies for improving HRQOL in SLE children**.**

**Supplementary Information:**

The online version contains supplementary material available at 10.1007/s10995-023-03680-x.

## Significance

This is the first study to evaluate HRQOL scores in Egyptian children with SLE which were found to be lower than published data with long disease duration, high cumulative disease activity, use of steroids and presence of obesity are the main influential factors related to low QoL scores. Generic and disease specific questionnaires are easily used for Arabic speaking subjects and easily interpreted by physician and can be implemented for frequent monitoring of SLE HRQOL. Controlling the disease activity and using lowest doses of steroids and other immunosuppressive drugs are the corner stone strategies for improving HRQOL in SLE children.

**Supplementary Information:**

The online version contains supplementary material available at 10.1007/s10995-023-03680-x.

## Introduction

Childhood Systemic lupus erythematosus (cSLE) is characterized by severe course, widespread organ involvement especially renal and central nervous systems (CNS) and high mortality compared to adult-onset SLE (Ravelli et al., 2005). Young adults with SLE reported to have mortality rates 20- times higher than general population (Brunner et al., 2008). With the advances in treatment of cSLE, mortality rates decreased and life expectancy of the patients increased associated with a wide-ranging spectrum of long-term morbidities which accumulates more quickly in cSLE compared to adult-onset caused by higher rates of nephritis (Levy et al., 2014), more aggressive course which increases the exposure to immunosuppressive medications over a longer disease duration and the impact of disease and medications side effects on school performance and later on sexual and reproductive lives of affected adolescents (Hersh et al., 2009).

Health- related quality of life (HRQOL) has been defined as a “multi-dimensional concept including physical, social and psychological functioning related to a certain illness or its treatment” (Miller et al., 2002). The physical, emotional, and social effects of SLE drastically influence the QoL of patients. This can be related to repeated hospitalizations, follow up visits, frequent laboratory monitoring, health-care costs, and interference with daily activities (Hersh et al., 2009; Mina & Brunner, 2010). Research in adults with SLE suggests that the disease has a significant negative impact on the patients’ HRQOL (Thumboo & Strand, 2007). Similar research in children is lacking as there are few studies examining the HRQOL in small cSLE cohorts using standardized instruments (Brunner et al., 2009; Moorthy et al., 2017).

So, evaluating the different domains of QoL of children with SLE using pediatric specific tools have become critical determinants of the disease impact (Brunner & Giannini, 2003; Moorthy et al., 2005; Ravelli et al., 2005). Considering the paucity of information of QoL in cSLE in general, and Egyptian children particularly, we conducted the present study. The objectives of our study were to assess the QoL in children aged 8–18 years with SLE using the Pediatric Quality of Life Inventory Generic Core Scales (PedsQL™ 4.0 GCS), the PedsQL™ 3.0 Rheumatology Module (PedsQL™ 3.0 RM) and the Simple Measure of the Impact of Lupus Erythematosus in Youngsters (SMILEY), compare it to that of available cohorts; and assess the relationship of QoL with SLE disease features, activity, and damage scores.

## Subjects and Methods

### Participants

This questionnaire-based study was conducted in Mansoura University Children’s Hospital in Egypt over the period of 12 months. The study was approved by the local ethical committee and all participants/ guardians gave their informed consent prior to their inclusion in the study. A standardized introduction on HRQOL was given to both patients and parents prior to fulfilment of the questionnaires.

#### Inclusion Criteria

All included children were diagnosed to have SLE after fulfilling the American College of Rheumatology (ACR) classification criteria (Hochberg, 1997) and with a duration of illness of more than 1-year, normal kidney function, with no active infection and not admitted to the hospital at time of enrolment in the study.

#### Exclusion Criteria

Children with disabilities, psychological problems or obesity that have been diagnosed before the onset of SLE and those with mixed connective tissue diseases were excluded.

During the period of the study and after applying inclusion and exclusion criteria, 123 cSLE patients were approached to participate of which 100 were enrolled (23 patients not included as either refused to participate or agreed initially then did not return completed questionnaire form).

All patients with clinically evident lupus nephritis (LN) had ultrasound guided renal biopsy before starting treatment. Total cumulative steroid dose was calculated for patients’ weights and presented as g/kg according to which patients were divided into 2 groups: high cumulative glucocorticoids (CGCS) dose (> 1 gm/kg) and low CGCS (< 1 gm /kg). Obesity was defined as body mass index (BMI) above 95th percentile for age. Bone complications included: pathological fracture, avascular necrosis of femoral head and low bone mineral density (BMD) diagnosed as BMD z-score < -2 (El-Ziny et al., 2007).

Current and previously reported disease activity [evaluated by SLE Disease Activity Index 2000 (SLEDAI-2 K)] (Gladman et al., 2002) and disease related damage evaluated by SLE International Collaborating Clinics/ American College of Rheumatology Damage Index (SDI) were also obtained. SDI is an instrument that allows evaluation of accumulated damage in SLE patients including evaluation of 12 organ systems. Damage may be caused by previous disease activity or by medications and should be ascertained by clinical assessment and present for at least 6 months. A higher SDI score indicates worsening disease damage (Gladman et al., 1996). For comparative purposes, we used cutoff values like published articles to group the patients with greater disease activity and damage (cutoffs for SDI ≥ 2, SLEDAI ≥ 12) (Moorthy et al., 2007, 2009, 2017).

### Procedures

All questionnaires were completed in paper form only. Questionnaire forms were completed during clinic visits. Questionnaires were completed by the children, with only few patients required some help from parents to understand some question (without guiding their answers).

Data were collected by a structured questionnaire using PedsQL**™** 4.0 GCS, the PedsQL™ 3.0 RM, and the SMILEY module. An “Arabic for Egypt” version (Language of all participants) of the PedsQL**™** 4.0 GCS is available for free after registration at (https://eprovide.mapi trust.org/instruments/pediatric-quality-of-life-inventory# need _ this _ questionnaire) provided by MAPI research institute, Lyon, France]. This version of the questionnaire has been previously used in the assessment of QoL in Egyptian children with nephrotic syndrome (NS) (Eid et al., 2020). The PedsQL™ 3.0 RM was translated into Arabic by the authors, and a professional English language expert. The Arabic copy of the questionnaire was approved by MAPI research institute, France and by professor James W. Varni. A free Arabic “for Egypt” copy of the SMILEY module was provided by Professor Moorthy with the permission to use it.

### Measures

#### PedsQL™4.0 Generic Core Scale

We used the children (8–12 years) and adolescents (13–18 years) copies of the questionnaire according to patients’ age. A 5-point response scale was used from 0 to 4. Items are reverse-scored and converted to a 0–100 scale so higher scores indicate better QoL. Scale scores are computed as the sum of the items divided by the number of items answered (accounting for missing data). If more than 50% of the items in the scale are missing, the scale score is not computed (Varni et al., 2001). This accounts for the differences in sample sizes for scales reported in the results’ tables**.** The obtained results in all domains of PedsQL™ 4.0 were then compared to its previously published normative data (Varni et al., 2003) and results of 100 healthy control Egyptian children published in 2020 (Eid et al., 2020).

#### The PedsQL™ 3.0 RM

We used the children (8–12 years) and adolescents (13–18 years) copies of the questionnaire. The tool evaluates the QoL in 5 domains (pain and hurt, daily activities, treatment, worry, and communication). The format, instructions, Likert scale, and scoring scheme are the same as the PedsQL™ 4.0, with higher scores indicating better QoL (Varni et al., 2001). For our study, we used PedsQL™ 3.0 RM ratings of a cohort of 56 children with juvenile idiopathic arthritis (JIA) as normative values for comparison which was the first study to validate this rheumatology specific questionnaire in children with JIA being the most common pediatric rheumatology disorder (Brunner et al., 2004).

#### SMILEY

The SMILEY is the only disease-specific measure of HRQOL in cSLE. We used child report of the SMILEY measure. The SMILEY is a 26-item survey. The first 2 survey items are summary questions that are not included in the final score. Item 1 relates to current HRQOL status and item 2 relates to current SLE status. Responses are in the form of an illustrative 5-step scale with different facial expressions. If more than 12 questions are not answered, SMILEY cannot be scored. All items, including the first 2 summary questions, score from 1 to 5. The total score is transformed to a 1–100 scale (according to the scoring manual provided by Professor Moorthy). Higher scores reflect better QoL. Being a disease specific tool, there are no normative values for the SMILEY questionnaire (Moorthy et al., 2006).

### Statistical Analysis

A sample size of 100 cSLE patients was chosen based on the duration of the study, availability of cases that fulfil inclusion criteria and collaboration of patients. Continuous variables with normal distribution were expressed as means and standard deviation and compared using Student’s t-test, whereas those not normally distributed were using Mann–Whitney U-test. Categorical variables were compared using the Chi-square test or Fisher’s exact test. Subsequently, the association of demographic parameters, clinical features, and disease complications to QoL scores was investigated for SLE patients (Table 6) using Pearson correlation or t-test (compare means) as relevant to the included variable. Correlation between SMILEY scores and each of PedsQL™ 4.0 GCS and PedsQL™ 3.0 RM scores was conducted. Statistical analysis was done using Statistical Package for the Social Science software version 25.0 (SPSS Inc., Chicago, IL, USA).

## Results

One hundred children with SLE participated in the study, 79 females, and aged 14.04 ± 2.5 years at time of the study. Characteristics of patients are summarized in Table 1. All SLE children had normal kidney function at enrollment with eGFR 140.7 ± 25.8 ml/min/1.73^2^. The number of medications taken by each patient at enrolment ranged between 2 and 5 medications including steroids, hydroxychloroquine, mycophenolate mofetil (MMF), cyclosporine, cyclophosphamide, antihypertensives, calcium and vitamin supplementation. Due to presence of missing data and as described in methodology section, total scores of the 3 used measures were calculated for 98 patients each. The mean scores for each domain and summary score of the 3 scales are presented in Tables 2 and 3. The mean scores for PedsQL™ 4.0 are compared to published normative data (Varni et al., 2003) and to the scores of 100 healthy Egyptian children data published in 2020 (Eid et al., 2020) showing significantly lower scores for SLE patients in all domains. The mean scores for PedsQL3-RM are compared to children diagnosed with JIA as a comparative population.

**Table 1:** Clinical and laboratory data of the patients

Parameter	Results
Sex male/female	21/79
Age at diagnosis(years) mean ± SD	11.5 ± 2.6
Duration of illness (months) mean ± SD, range	31.4 ± 19.9, 12–108
Residence rural/urban	66/34
School level (primary/preparatory/secondary/non-educated)	20/36/43/1
Family size range	(3–9)
(Up to 5/above 5)	70/30
Source of medications:	
Self-purchased	3
Hospital	65
Health insurance	7
Mixed	25
Clinical manifestations (number/percentage):	
LN (class I, II, III, IV, V, VI)	70(8, 11, 23, 27, 1, 0)
Skin manifestations	75
CNS	11
Arthritis	64
Serositis	18
Number of hospital admissions (range), mean ± SD	(0–9) 1.6 ± 1.6
SLEDAI score: mean ± SD	
At enrolment:	7.5 ± 4.6
Cumulative:	8.3 ± 3.9
SDI: at enrolment: mean ± SD	0.42 ± 0.85
BMD Z-score mean ± SD	− 0.65 ± 1.3
Medications	
Steroid + hydroxychloroquine only	42
Cyclophosphamide (ever)	40
MMF	12
Azathioprine	7
CGCS: mean ± SD	0.84 ± 0.61
High/low	37/63
Complications	
Decreased BMD (Z-score < -2)	19
Pathological fracture/AVN	3
Alopecia	47
Cushingoid manifestations	35
Diabetes Miletus	2
Ocular complications	0
Obesity	33
Hypertension	15

*SLE* Systemic lupus erythematosus, *SDI* SLE damage index, *SLEDAI* SLE disease activity index, *BMD* bone mineral density, *AVN* avascular necrosis, *MMF* Mycophenolate mofetil, *SD* standard deviation, *CNS* central nervous system, *LN* lupus nephritis, *CGCS* cumulative glucocorticoids

**Table 2:** Comparison of PedsQL™ 4.0 and PedsQL™ 3.0 RM results and normative data

Measure	Normative data mean ± SD (N)		SLE patients mean ± SD (N)	*p*-value	
	Published Normative	Egyptian children			
*PedsQL™ 4.0 GCS*					
Summary score	82.9 ± 13.2(5972)	86.02 ± 6.1 (96)	71.3 ± 11.6(98)	< 0.001**	< 0.001***
Physical	86.9 ± 13.9(5962)	87.3 ± 8.6(99)	69.3 ± 13.5(99)	** < 0.001**	** < 0.001**
Emotional	78.2 ± 18.6(5961)	87.3 ± 7.5 (99)	72.3 ± 13.7(98)	**0.002**	** < 0.001**
Social	84.0 ± 17.4(5948)	86.7 ± 7.7(99)	71.2 ± 12.9(99)	** < 0.001**	** < 0.001**
School	79.9 ± 16.9(5908)	86.45 ± 7.9(99)	71.6 ± 11.9(97)	** < 0.001**	** < 0.001**
PedsQL™ RM*	Published normative (N = 56)				
Summary score	84.4 ± 18		72.1 ± 9.9(98)	** < 0.0001**	
Daily activities	95.6 ± 11.1		71.9 ± 15.1(99)	** < 0.0001**	
Treatment	82.1 ± 16.3		78.4 ± 11.9(99)	0.1	
Pain and hurt	77.8 ± 24.2		74.2 ± 14.2(97)	0.2	
Communication	87.9 ± 16.3		73.2 ± 8.3(100)	** < 0.0001**	
Worry	83.6 ± 18.5		62.8 ± 9.8(98)	** < 0.0001**	

*PedsQL™ 4 GCS* Pediatric Quality of Life Inventory Generic Core Scales, *PedsQL™ 3.0 RM* the PedsQL™ 3.0 Rheumatology Module, *SLE* systemic lupus erythematosus, *SD* standard deviation

*Source for PedsQL RM norms in 56 children with juvenile rheumatoid arthritis

**SLE patients vs published normative data

***SLE patients vs Egyptian children control data

Bold figures are the statistically significant values

**Table 3:** Comparison of SMILEY scores of current study and published studies

Domain	Study	Results	Current study results	p-value
		Mean ± SD(N)	Mean ± SD(N)	95%CI
Total	Moorthy et al. (2009)* USA Mixed ethnicity	64 ± 12 (67)	58.8 ± 19.4(98)	0.05 (− 10.5 to 0.06)
Effect on self		64 ± 15(66)	63.5 ± 24.3(96)	0.9(− 7.1 to 6.1)
Limitations		63 ± 16(65)	58.3 ± 21.9(96)	0.14(− 10.9 to 1.56)
Effect on social life		81 ± 16(67)	73.3 ± 21.3(96)	**0.01(− 13.7 to − 1.6)**
Burden of SLE		58 ± 15(65)	53.7 ± 25.1(98)	0.22(− 11.1 to 2.5)
Total	Moorthy et al. (2017)* North America	65 ± 14(182)	58.8 ± 19.4(98)	**0.002(− 10.2 to − 2.2)**
Effect on self		65 ± 17(181)	63.5 ± 24.3(96)	0.55(− 6.4 to 3.4)
Limitations		64 ± 17(182)	58.3 ± 21.9(96)	**0.02(− 10.4 to − 1.02)**
Effect on social life		82 ± 16(182)	73.3 ± 21.3(96)	** < 0.001(− 13.2 to − 4.2)**
Burden of SLE		57 ± 17(182)	53.7 ± 25.1(98)	0.2(− 8.3 to 1.7)
Total	Moorthy et al. (2017)* South America	67 ± 15(106)	58.8 ± 19.4(98)	** < 0.001(− 12.9 to − 3.4)**
Effect on self		68 + 20(106)	63.5 ± 24.3(96)	0.15(− 10.7 to 1.7)
Limitations		66 ± 19(106)	58.3 ± 21.9(96)	**0.008(− 13.3 to − 2.02)**
Effect on social life		84 ± 15(106)	73.3 ± 21.3(96)	** < 0.0001(− 15.8 to − 5.6)**
Burden of SLE		56 ± 19(106)	53.7 ± 25.1(98)	0.5(− 8.4 to 3.8)
Total	Moorthy et al. (2017)* Europe	68 ± 16(78)	58.8 ± 19.4(98)	** < 0.001(− 14.6 to − 3.8)**
Effect on self		68 ± 20(78)	63.5 ± 24.3(96)	0.19(− 11.3 to 2.3)
Limitations		66 ± 20(73)	58.3 ± 21.9(96)	0.02(− 14.2 to − 1.2)
Effect on social life		86 ± 15(77)	73.3 ± 21.3(96)	** < 0.0001(− 18.4 to − 7.03)**
Burden of SLE		60 ± 18(78)	53.7 ± 25.1(98)	0.06(− 12.9 to 0.36)
Total	Moorthy et al. (2017)* Asia	72 ± 14(86)	58.8 ± 19.4(98)	** < 0.0001(− 18.2 to − 8.2)**
Effect on self		72 ± 17(87)	63.5 ± 24.3(96)	**0.007(− 14.6 to − 2.3)**
Limitations		71 ± 17(86)	58.3 ± 21.9(96)	** < 0.0001(− 18.5 to − 6.9)**
Effect on social life		86 ± 14(86)	73.3 ± 21.3(96)	** < 0.0001(− 18.03 to − 7.4)**
Burden of SLE		62 ± 19(78)	53.7 ± 25.1(98)	**0.02(− 15.1 to − 1.5)**
Total	Moorthy et al. (2013)* Brazil	67 ± 16(93)	58.8 ± 19.4(98)	**0.002(− 13.3 to − 3.1)**
Effect on self		70 ± 20(93)	63.5 ± 24.3(96)	**0.05 (− 12.9 to − 0.1)**
Limitations		66 ± 20(93)	58.3 ± 21.9(96)	**0.01(− 13.7 to − 1.7)**
Effect on social life		85 ± 14(93)	73.3 ± 21.3(96)	** < 0.0001(− 16.9 to − 6.5)**
Burden of SLE		57 ± 19(93)	53.7 ± 25.1(98)	0.3(− 9.6 to 3.1)

*First visit child report results only are included in comparison

*SMILEY* Simple Measure of the Impact of Lupus Erythematosus in Youngsters, *SLE* systemic lupus erythematosus

Bold figures are the statistically significant values

(Brunner et al., 2004) (Table 2) and showed significantly lower scores for in SLE patients compared to controls in all domains except for treatment and pain and hurt domains. Being a disease specific scale, SMILEY has no normative data, so we compared our results to published studies from different countries (Table 3).

As skin manifestations and LN are the most prevalent manifestations and LN being a leading cause of morbidities and mortalities and a unique feature of cSLE compared to other pediatric rheumatologic disorders and adult onset SLE, the total and domains’ scores for the used measures were compared between patients with and without LN and showed that SMILEY total and domain scores were significantly lower in patients with LN compared to those without LN, while no significant differences in PedsQL™ 4.0 scores and only worry domain of PedsQL3-RM was significantly lower in LN patients. Regarding skin manifestations, no significant differences reported in total and all domain scores of the 3 scales in patients with and without lupus skin manifestations**.** Disease activity was evaluated by SLEDAI score according to which patients were classified as having no/mild (SLEDAI < 12) (N = 77) or severe disease activity (SLEDAI ≥ 12((N = 23). The mean scores for all domains of the 3 scales for the 2 groups (mild and severe active diseases) were calculated and compared in Table 4. Disease related damage was evaluated by SDI according to which patients were classified as having no or minimal damage (SDI < 2) (N = 92) or severe damage (SDI ≥ 2) (N = 8). The mean scores for all domains of the 3 used scales for the 2 groups (no/mild and severe severe) were calculated and compared in Table 5. Table 6 shows correlation of QoL scores of the 3 measures used and different clinical manifestations and revealed that longer duration of illness, high cumulative steroid doses, higher SLEDAI and SDI scores, presence of obesity and low BMD were associated with low QoL scores for all 3 tools (*p* < 0.001). No significant differences were reported between male and female patients scores of all domains of 3 scales, disease activity or damage (*p* > 0.05 for all). Spearman correlation showed that SMILEY total scores corelated more strongly with PedsQL3-RM (*r* = 0.917, *p* < 0.001) than PedsQL™ 4.0 (*r* = 0.753, *p* < 0.001).

**Table 4:** Quality of life scores in patients with low versus high disease activity

Measure	Low disease activity (N = 77)	High disease activity (N = 23)	*P*-value
	SLEDAI < 12	SLEDAI ≥ 12	
	Mean ± SD(N)	Mean ± SD	
*PedsQL™ 4.0 GCS*			
Summary score	73.7 ± 10.35(75)	63.8 ± 12.2(23)	** < 0.001**
Physical	71.5 ± 12.5(77)	61.3 ± 14.2(22)	**0.002**
Emotional	74.9 ± 12.58(75)	64.04 ± 14.2(23)	**0.001**
Social	73.3 ± 11.5(76)	64.13 ± 14.7(23)	**0.002**
School	73.6 ± 10.25(75)	65 ± 14.56(22)	**0.002**
PedsQL™ 3 RM*			
Summary score	73.86 ± 9.6(76)	65.87 ± 8.98(22)	**0.001**
Daily activities	72.8 ± 16.1(77)	68.7 ± 11.1(22)	0.3
Treatment	80.3 ± 11.8(77)	71.8 ± 9.89(22)	**0.003**
Pain and hurt	76.7 ± 13.1(75)	65.6 ± 14.7(22)	**0.001**
Communication	75.1 ± 7.68(77)	67.1 ± 7.49(23)	** < 0.001**
Worry	64.4 ± 9.96(75)	57.8 ± 7.4(23)	**0.004**
*SMILEY*			
Total	62.4 ± 18.59(76)	46.1 ± 16.79(22)	** < 0.001**
Effect on self	67.4 ± 23.7(75)	50.2 ± 22.14(22)	**0.003**
Limitations	62.5 ± 29.87(75)	43.5 ± 19.6(21)	** < 0.001**
Effect on social life	77.09 ± 19.3(74)	60.68 ± 23.3(22)	**0.001**
Burden of SLE	57.4 ± 21.2(75)	41.86 ± 21.2(23)	**0.009**

*PedsQL™ 4 GCS* Pediatric Quality of Life Inventory Generic Core Scales, *PedsQL™ 3.0 RM* the PedsQL™ 3.0 Rheumatology Module, *SLE* systemic lupus erythematosus, *SD* standard deviation, *SLEDAI* SLE disease activity index

Bold figures are the statistically significant values

**Table 5:** Quality of life scores in patient with and without damage

Measure	Minimal or no damage (N = 67)	Damage (N = 23)	*p*-value
	SDI ≤ 2	SDI > 2	
	mean ± SD (N)	mean ± SD(N)	
*PedsQL™ 4.0 GCS*			
Summary score	72.2 ± 11.6(90)	61.4 ± 4.6(8)	**0.01**
Physical	70.7 ± 13.2(91)	53.4 ± 0.8(8)	** < 0.001**
Emotional	72.8 ± 13.9(90)	67.3 ± 10.8(8)	0.3
Social	72.3 ± 12.7(91)	58.8 ± 6.4(8)	** < 0.001**
School	72.1 ± 11.9(89)	66.9 ± 9.6(8)	0.2
PedsQL™ 3 RM*			
Summary score	73.1 ± 9.8(90)	60.6 ± 3.1(8)	**0.001**
Daily activities	72.6 ± 15.5(91)	64.3 ± 6.7(8)	0.14
Treatment	79.5 ± 11.6(91)	66.3 ± 8.3(8)	**0.002**
Pain and hurt	75.7 ± 13.9(89)	57.8 ± 3.2(8)	** < 0.001**
Communication	74.1 ± 7.9(92)	63.3 ± 4.8(8)	** < 0.001**
Worry	63.6 ± 9.6(90)	51.5 ± 1.8(8)	** < 0.001**
*SMILEY*			
Total	61.1 ± 18.5(90)	33.2 ± 3.6(8)	** < 0.001**
Effect on self	66.2 ± 23.6(89)	33.5 ± 4.7(8)	** < 0.001**
Limitations	60.4 ± 21.7(88)	35.3 ± 7.1(8)	** < 0.001**
Effect on social life	76.5 ± 19.2(88)	38.1 ± 8.4(8)	** < 0.001**
Burden of SLE	56.1 ± 24.8(90)	27.5 ± 1.5(8)	** < 0.001**

*PedsQL™ 4 GCS* Pediatric Quality of Life Inventory Generic Core Scales, *PedsQL™ 3.0 RM* the PedsQL™ 3.0 Rheumatology Module, *SLE* systemic lupus erythematosus, *SD* standard deviation, *SDI* systemic lupus damage index

Bold figures are the statistically significant values

**Table 6:** Analysis of possible associations of QoL Summary scores with different clinical and laboratory data of SLE patients

Parameter	PedsQL™ 4.0 scores	PedsQL™ 3.0 RM scores	SMILEY
	*p-*value, r*	*p-*value, r	*p-*value, r
Age at enrolment	**0.004, − 0.288**	**0.009, − 0.262**	**0.004, − 0.286**
Age at diagnosis	0.77, − 0.03	0.5, 0.065	0.8, − 0.023
Duration of illness	** < 0.001, − 0.527**	** < 0.001, − 0.606**	** < 0.001, − 0.539**
Number of hospital admissions	0.3, − 0.108	0.08, − 0.175	**0.004, − 0.289**
Number of medications	0.4, − 0.094	**0.007, − 0.284**	**0.002, − 0.32**
SLEDAI score			
Current	**0.001, − 0.34**	** < 0.001, − 0.4**	** < 0.001, − 0.453**
Cumulative	**0.04, − 0.207**	**0.03, − 0.223**	**0.01, − 0.257**
SDI score	** < 0.001, − 0.352**	** < 0.001, − 0.395**	** < 0.001, − 0.451**
	***p-*value (95% CI)**	***p-*value (95% CI)**	***p-*value (95% CI)**
Sex: male vs female	0.2 (3.7 to 2.8)	0.09 (− 0.7- 9.1)	0.44 (− 5.7 to 13.6)
Geographic location: Rural vs urban	0.13 (− 8.6 to 1.13)	**0.01 (− 9.5 to − 1.3)**	**0.008 (− 18.7 to − 2.9)**
Family size: below 5 vs above 5	0.7 (− 4.02 to 6.2)	0.3 (2.2 to 2.2)	0.7(− 7.07 to 10)
Renal involvement: nephritis vs non-nephritis	0.5 (− 6.9 to 3.2)	0.2 (− 7.4 to 1.2)	0.01 (− 19.2 to − 2.3)
Arthritis: + ve vs − ve	0.4 (− 7.8 to 3.3)	0.4 (− 2.4 to 6.5)	0.2 (− 3.2 to 15)
CGCS: high vs low	**0.002 (2.7 to 11.9)**	** < 0.001 (5.8 to 13.2)**	**0.001 (5.7 to 21.1)**
Obesity yes vs no	**0.002 (− 12.3 to − 2.9)**	** < 0.001 (− 13.4 to − 5.8)**	** < 0.001 (− 22 to − 6.4)**
Reduced BMD: + ve vs _ve	** < 0.001 (− 16.6 to − 5.4)**	** < 0.001 (− 14.5 to − 4.8)**	** < 0.001 (− 28.7 to − 10.7)**
Alopecia yes vs no	0.9 (− 4.5 to 4.9)	0.7 (− 4.9 to 3.2)	**0.02 (− 16.7 to − 1.4)**
Hypertension: yes vs no	0.2 (− 9.4 to 2.2)	0.06 (− 9.9 to 0.29)	**0.02 (− 21.8 to − 2.3)**
Cyclophosphamide use (ever) yes vs no	0.6 (− 5.9 to 3.5)	0.08 (− 7.6 to 0.5)	**0.01 (− 17.8 to − 2.4)**

*PedsQL™ 4 GCS* Pediatric Quality of Life Inventory Generic Core Scales, *PedsQL™ 3.0 RM* the PedsQL™ 3.0 Rheumatology Module, *SLE* systemic lupus erythematosus, *SD* standard deviation, *SDI* systemic lupus damage index. *CGCs* cumulative glucocorticoids, *SLEDAI* SLE disease activity index, *BMD* bone mineral density

*r Pearson correlation coefficient

Bold figures are the statistically significant values

## Discussion

The differences in cSLE regarding disease onset, phenotype, and outcomes between different ethnic groups is not fully elucidated (Massias et al., 2021). Additionally, differences in health care levels and attention to Qol also differ between countries. In Egypt the overall estimated prevalence of cSLE is 1/100,000 population (0.24/100000 males and 1.8/100000 females). The age of onset and female predominance are like other countries however, LN is more common in Egypt compared to cSLE from other countries (Eesa et al., 2021) which is associated with use of more immunosuppressives, frequent hospitalization and risk of deterioration of kidney functions. Youth with SLE in Egypt face many challenges including late diagnosis, cost of diagnostic work up, and treatment. Moreover, pediatric rheumatology/nephrology service is available only in few centers in Egypt. Some medications including recent biologics are not available in Egypt which limit choices available for refractory cases. Cyclophosphamide is used in up to 50% of cSLE in Egyptian patients due to its availability and low cost despite being not preferred by the patients and families due to cosmetic and gonadal side effects (Eesa et al., 2021 and Elmougy et al., 2015). Qol is not part of the care of youth with SLE in Egypt despite all the challenges they are facing. This was the motive for conducting the current study.

Multiple scales have been used to evaluate QoL in cSLE (Levy et al., 2014; Putera et al., 2020). Regarding generic questionnaires, Varni et al;(2001) reported that PedsQL™ 4.0 summary score in SLE was significantly lower than healthy children and that PedsQL™ 4.0 can differentiate between healthy children and those with rheumatology disorder. Brunner et al; (2009) reported that CHQ-P50 and the PedsQL™ 4.0 scores of SLE children were lower than healthy children and that the 2 measures are responsive to clinically important changes in cSLE disease activity**.** In the current study, all domains of the PedsQL™ 4.0 were significantly lower in SLE children compared to published normative data (Varni et al., 2003) and to a previously published data of healthy Egyptian children (Eid et al., 2020) but with no difference in affection between domains which is also inconsistent with Rogers et al. who reported school domain to be the most affected (Rogers et al., 2017) and also a report from USA (Stevens et al., 2019). Noticeably, all domains of PedsQL™ 4.0 of our patients are lower than scores of SLE children reported by Brunner et al., (2009), Stevens et al; (2019) but not different from earlier reports as Varni et al; (2002) (except for social domain). The inconsistency of the results of the studies may be due to differences in selection criteria of patients, ethnic differences which modify disease presentation and severity, differences in patients’ care between countries and the improvement of patients’ care over years.

Multiple rheumatology specific QoL questionnaires are available and some of them have been used for SLE children (Duffy, 2005; Duffy et al., 1997; Wright et al., 1994) with PedsQL™ 3.0 RM introduced by Varni et al; (2002) being the most popular. In the current work, despite having a total PedsQL™ 3.0 RM score above 70 which is recognized to be good QoL score as stated by Varni et al; (2001), the score is still significantly lower than normative data of children with JIA (Brunner et al., 2004) with the worry domain scores being the lowest in our cohort. Comparing our results to the report by Stevens et al; (2019), total and all domain scores of PedsQL™ 3.0 RM were significantly lower in our cohort. Putera et al; (2020) reported PedsQL™ 3.0 RM scores to be much lower in children with LN during induction phase compared to maintenance phase**.** This again emphasis that that characteristics of patients at time of enrolment in the study have great effect on the scores of HRQOL questionnaires and could account for the inconsistency of results between studies despite using same scales.

In pediatric rheumatology practice, outcome measures are primarily concentrated on the impact of physical disability pain on different aspects of life, as most of the children have JIA. QoL measures that have been developed for children with chiefly musculoskeletal involvement may not adequately assess all aspects of illness in cSLE. Although physical function is an important domain affected in SLE, a true measure of SLE-related QoL must take into consideration other domains of HRQOL and adequately assess the impact of renal and CNS involvement, as well as the effect of medications (Moorthy et al., 2005). So, SLE-specific QoL tool titled (SMILEY^©^) was developed by Moorthy et al; (2006) and have been validated in different languages including Arabic (Moorthy et al., 2006, 2007). Being a disease-specific scale, SMILEY has no normative data, so we matched our results to previously published SMILEY results from different countries as presented in Table 3 which showed variable results in the different domains. The comparatively high HRQOL among Asian patients could be due to cultural factors, family support, provider preferences related to medication choice that led to disease control or differences in health care systems (Moorthy et al., 2017). Characteristics of patients at time of enrolment in study is also a critical determinant of the questionnaires’ scores. Moorthy et al. (2017) reported the median SLEDAI and SDI scores significantly lower than our cohort. Also, the Brazilian cohort (Moorthy et al., 2013) had a duration of illness 1–153 months and median SLEDAI and SDI scores of 2 and zero respectively which are lower than the current study.

Childhood SLE is more frequent in females which is also spotted in our study and consistent with previous reports from our country and others (Brunner et al., 2009; Eid et al., 2021; Ruperto et al., 2004). Moorthy et al. reported female gender to be associated with lower QoL scores (Moorthy et al., 2017), which was not reported in our study. Patients with LN showed lower scores for SMILEY scale compared to those without LN, but similar finding was not reported in the other 2 scales used. This supports the assumption that SMILEY being disease specific has better ability to evaluate QoL of children with SLE. The relationship of HRQL with renal manifestations, which are generally associated with a worse clinical course and prognosis (Petty & Cassidy, 2001), may reflect a tendency for patients with a progressive disease to report lower HRQL, even if presented with nonthreatening manifestations. The poorer HRQOL in patients with renal and CNS involvement could be related to the aggressive immunosuppressive regimens and higher doses of steroids which change body appearance and lead to impairment of self-confidence especially in adolescents (Ruperto et al., 2004). Patients who received cyclophosphamide had significantly lower SMILEY score than those who didn’t receive it. These findings are coherent with report of Moorthy et al. of 456 children with SLE (Moorthy et al., 2017). Arthritis which is a common feature in SLE was not associated with lower scores of the 3 scales used in the current study. This is against Stevens et al. (2019) who reported arthritis to be the feature affecting all QoL domains. Synovitis and arthritis are typical features of most pediatric rheumatic disorders, but the localization and the extent of the joints inflammation and erosions differs. Arthritis in SLE has short duration, good response to treatment, erosive arthritis is unusual and only a minority of patients develop deformities which was not reported in any of our patients.

Lower disease activity and damage were associated with higher QoL scores of the 3 scales (except for daily activities domain of PedsQL™ 4.0). These findings are consistent with most of published studies (Brunner et al., 2009; Moorthy et al., 2017; Ruperto et al., 2004) except Jones et al. (2013) who reported no such correlation that was explained that traditional measures of cSLE activity do not capture HRQOL outcomes adequately which may limit achievement of optimal health outcomes with cSLE. However, some features and complications of the disease that may also occur in many other chronic illnesses correlated significantly with QoL scores of all 3 scales as long duration of illness, high CGCS, obesity and decreased BMD. The main characteristics and findings of available studies on HRQOL in children with SLE are summarized in Supplementary Table 1.

This study explores for the first time HRQOL in Egyptian children with SLE which was found to be low. Our next step will be the evaluating parents view of their children life quality using the parents’ versions of the questionnaires used in the study which will add to the overall understanding of cSLE Qol in Egyptian youth. Next, Qol assessment will be implemented as part of the routine care and follow up of those children with a psychiatric/ social worker added to our pediatric rheumatology/nephrology care team for SLE children. Focus should be towards controlling disease activity using lowest doses and durations of medications, close monitoring of organ involvements and life quality scores.

Limitations of the current work is being a single center study, the lack of reference normative Egyptian data for the PedsQL™ 3.0 RM and the need for follow up evaluation of HRQOL scores changes over longer follow up duration and the evaluation of family impact scores.

## Conclusion

Quality of life assessment in Egyptian children with SLE is not yet included as a part of the standard care provided to these children despite being low as evaluated in the current work. Both generic and disease- or system-specific scales can be used for its assessment. Collaborative efforts from pediatrician, rheumatologists, and psychiatrics are urgently required to support those children.

## Supplementary Information

Below is the link to the electronic supplementary material.

Supplementary file1 (DOCX 41 KB)

## Data Availability

Data and material are available upon request.

## Abbreviations

**ACR**: American college of rheumatology
**BMD**: Bone mineral density
**BMI**: Body mass index
**CGCS**: Cumulative glucocorticoids
**cSLE**: Childhood systemic lupus erythematosus
**CNS**: Central nervous system.
**eGFR**: Estimated Glomerular filtration rate
**HRQOL**: Health related quality of life
**JIA**: Juvenile idiopathic arthritis
**LN**: Lupus nephritis
**MMF**: Mycophenolate mofetil
**QoL**: Quality of life
**PedsQL****™**** 4.0 GCS**: Pediatric Quality of Life Inventory Generic Core Scales
**PedsQL3-RM**: PedsQL™ 3.0 Rheumatology Module
**SLE**: Systemic lupus erythematosus
**SLEDAI**: Systemic lupus erythematosus disease activity index
**SDI**: SLE International Collaborating Clinics/ American College of Rheumatology Damage Index
**SMILEY**: Simple Measure of the Impact of Lupus Erythematosus in Youngsters

## Code Availability

Non applicable.

## References

[CR1] Brunner HI, Giannini EH (2003). Health-related quality of life in children with rheumatic diseases. Current Opinion in Rheumatology.

[CR2] Brunner HI, Klein-Gitelman MS, Miller MJ, Trombley M, Baldwin N, Kress A, Johnson AL, Barron AC, Griffin TA, Passo MH, Lovell DJ (2004). Health of children with chronic arthritis: Relationship of different measures and the quality of parent proxy reporting. Arthritis and Rheumatism.

[CR3] Brunner HI, Gladman DD, Ibañez D, Urowitz MD, Silverman ED (2008). Difference in disease features between childhood-onset and adult-onset systemic lupus erythematosus. Arthritis and Rheumatism.

[CR4] Brunner HI, Higgins GC, Wiers K, Lapidus SK, Olson JC, Onel K, Punaro M, Ying J, Klein-Gitelman MS, Seid M (2009). Health-related quality of life and its relationship to patient disease course in childhood-onset systemic lupus erythematosus. The Journal of Rheumatology.

[CR5] Duffy CM (2005). Measurement of health status, functional status, and quality of life in children with juvenile idiopathic arthritis: Clinical science for the pediatrician. Pediatric Clinics of North America.

[CR6] Duffy CM, Arsenault L, Duffy KN, Paquin JD, Strawczynski H (1997). The juvenile arthritis quality of life questionnaire–development of a new responsive index for juvenile rheumatoid arthritis and juvenile spondyloarthritides. The Journal of Rheumatology.

[CR7] Eesa NN, Abdel Nabi H, Owaidy RE, Khalifa I, Radwan AR, NourEl-Din AM, Amer MA, ElShereef RR, Hassan E, Ismail F, El-Gazzar II, Khalil NM, Moshrif AH, Abualfadl E, Tharwat S, Fathi HM, Abd Elazeem MI, El-Shebini E, Samy N, Noshy N, El-Bahnasawy AS, Abdalla AM, Abousehly OS, Mohamed EF, Nasef SI, Elsaman AM, ElKhalifa M, Salem MN, Abaza NM, Fathy HM, Abdel Salam N, El-Saadany HM, El-Najjar AR, El-Hammady DH, Hammam N, Mohammed RH, Gheita TA, Egyptian College of Rheumatology (ECR) SLE Study Group (2021). Systemic lupus erythematosus children in Egypt: Homeland spectrum amid the global situation. Lupus.

[CR8] Eid R, Fathy AA, Hamdy N (2020). Health-related quality of life in Egyptian children with nephrotic syndrome. Quality of Life Research.

[CR9] Eid R, Hammad A, Abdelsalam M, Fathy AA, Abd-El Ghafaar DM, Elmarghany EB, El-Hanafy AA, Mostafa N, Niazey NA, Korkor MS, Hamdy N (2021). Tumor necrosis factor receptor II and PTPN22 genes polymorphisms and the risk of systemic lupus erythematosus in Egyptian children. Lupus.

[CR10] Elmougy A, Sarhan A, Hammad A, El-Refaey A, Zedan M, Eid R, Laimon W, Elrahman AA, Elhussieni F, El-Sherbeny E, Bakr A (2015). Erratum to: Lupus nephritis in Egyptian children: A 16-year experience. Journal of Nephrology.

[CR11] El-Ziny MA, Al-Tonbary YA, Salama OS, Bakr A, Al-Marsafawy H, Elsharkawy AA (2007). Low bone mass in children with malignant lymphoma. Pediatric Hematology and Oncology.

[CR12] Gladman D, Ginzler E, Goldsmith C, Fortin P, Liang M, Urowitz M, Bacon P, Bombardieri S, Hanly J, Hay E, Isenberg D, Jones J, Kalunian K, Maddison P, Nived O, Petri M, Richter M, Sanchez-Guerrero J, Snaith M, Sturfelt G, Symmons D, Zoma A (1996). The development and initial validation of the Systemic Lupus International Collaborating Clinics/American College of Rheumatology damage index for systemic lupus erythematosus. Arthritis and Rheumatism.

[CR13] Gladman DD, Ibañez D, Urowitz MB (2002). Systemic lupus erythematosus disease activity index 2000. The Journal of Rheumatology.

[CR14] Hersh AO, von Scheven E, Yazdany J, Panopalis P, Trupin L, Julian L, Hersh AO, von Scheven E, Yazdany J, Panopalis P, Trupin L, Julian L, Katz P, Criswell LA, Yelin E (2009). Differences in long-term disease activity and treatment of adult patients with childhood- and adult-onset systemic lupus erythematosus. Arthritis and Rheumatism.

[CR15] Hochberg MC (1997). Updating the American College of Rheumatology revised criteria for the classification of systemic lupus erythematosus. Arthritis and Rheumatism.

[CR16] Houghton KM, Tucker LB, Potts JE, McKenzie DC (2008). Fitness, fatigue, disease activity, and quality of life in pediatric lupus. Arthritis and Rheumatism.

[CR17] Jones, J. T., Nelson, S. L., Wootton, J., Liberio, B., Greenler, A. J., Huggins, J. L., Huggins, J. L., Schanberg, L. E., & Brunner, H. (2013). Level and Determinants of Health-Related Quality of Life in Childhood-Onset Systemic Lupus Erythematosus, [Abstract 844]: 2013 ACR/ARHP Annual Meeting.

[CR18] Levy DM, Peschken CA, Tucker LB, Chédeville G, Huber AM, Pope JE, Canadian Network for Improved Outcomes in SLE (CaNIOS) 1000 Faces Investigators, & Silverman, E. D (2014). Association of health-related quality of life in childhood-onset systemic lupus erythematosus with ethnicity: results from a multiethnic multicenter Canadian cohort. Arthritis Care & Research.

[CR19] Massias JS, Smith EM, Al-Abadi E, Armon K, Bailey K, Ciurtin C, Davidson J, Gardner-Medwin J, Haslam K, Hawley DP, Leahy A, Leone V, McErlane F, Mewar D, Modgil G, Moots R, Pilkington C, Ramanan AV, Rangaraj S, Riley P, Sridhar A, Wilkinson N, Beresford MW, Hedrich CM (2021). Clinical and laboratory phenotypes in juvenile-onset systemic lupus erythematosus across ethnicities in the UK. Lupus.

[CR20] Miller ML, LeBovidge J, Feldman B (2002). Health-related quality of life in children with arthritis. Rheumatic Diseases Clinics of North America.

[CR21] Mina R, Brunner HI (2010). Pediatric lupus–are there differences in presentation, genetics, response to therapy, and damage accrual compared with adult lupus?. Rheumatic Diseases Clinics of North America.

[CR22] Moorthy LN, Peterson M, Onel KB, Harrison MJ, Lehman TJ (2005). Quality of life in children with systemic lupus erythematosus. Current Rheumatology Reports.

[CR23] Moorthy LN, Peterson MG, Baratelli M, Harrison MJ, Onel KB, Chalom EC, Haines K, Hashkes PJ, Lehman TJ (2006). Simple Measure of The Impact of Lupus Erythematosus In Youngsters (SMILEY): Validity, internal consistency and test-retest reliability. Arthritis and Rheumatism.

[CR24] Moorthy LN, Peterson MG, Baratelli M, Harrison MJ, Onel KB, Chalom EC, Haines K, Hashkes PJ, Lehman TJ (2007). Multicenter validation of a new quality of life measure in pediatric lupus. Arthritis and Rheumatism.

[CR25] Moorthy LN, Peterson MG, Hassett AL, Baratelli M, Chalom EC, Hashkes PJ, Hong S, Reiff A, Lehman TJ (2009). Relationship between health-related quality of life and SLE activity and damage in children over time. Lupus.

[CR26] Moorthy LN, Saad-Magalhães C, Sato JO, Len CA, Vasco MB, Appenzeller S, Marini R, Oliveira SK, Rodrigues M, Sztajnbok F, Almeida RG, Jesus AA, Campos LM, Silva CA, Peterson MG, Hassett AL, Weiss E, Verma S, Dahodwala MQ, Lehman TJ (2013). Validation of the Portuguese Simple Measure of Impact of Lupus Erythematosus in Youngsters (SMILEY) in Brazil. Lupus.

[CR27] Moorthy LN, Baldino ME, Kurra V, Puwar D, Llanos A, Peterson MG, Hassett AL, Lehman TJ, International SMILEY© Collaborative group (2017). Relationship between health-related quality of life, disease activity and disease damage in a prospective international multicenter cohort of childhood onset systemic lupus erythematosus patients. Lupus.

[CR28] Petty RE, Cassidy JT, Cassidy JT, Petty RE (2001). Systemic lupus erythematosus. Textbook of pediatric rheumatology.

[CR29] Putera AM, Irwanto I, Maramis MM, Prasetyo RV, Soemyarso NA, Noer MS (2020). Effect of mental health problems on the quality of life in children with lupus nephritis. Neuropsychiatric Disease and Treatment.

[CR30] Ravelli A, Ruperto N, Martini A (2005). Outcome in juvenile onset systemic lupus erythematosus. Current Opinion in Rheumatology.

[CR31] Rogers J, Sagcal-Gironella ACP, Rosillo P, Ramirez AA, Banuelos R, de Guzman MM (2017). A single center review of health-related quality of life in children with systemic lupus erythematosus using the pediatric quality of life inventory version 4.0 generic core scale. Arthritis & Rheumatology.

[CR32] Ruperto N, Buratti S, Duarte-Salazar C, Pistorio A, Reiff A, Bernstein B, Maldonado-Velázquez MR, Beristain-Manterola R, Maeno N, Takei S, Falcini F, Lepore L, Spencer CH, Pratsidou-Gertsi P, Martini A, Ravelli A (2004). Health-related quality of life in juvenile-onset systemic lupus erythematosus and its relationship to disease activity and damage. Arthritis and Rheumatism.

[CR33] Stevens B, Rodriguez M, Rakestraw A, O’neil K (2019). SAT0477 quality of life in subjects with pre-pubertal onset systemic lupus erythematosus. Annals of the Rheumatic.

[CR34] Thumboo J, Strand V (2007). Health-related quality of life in patients with systemic lupus erythematosus: An update. Annals of the Academy of Medicine, Singapore.

[CR35] Varni JW, Burwinkle TM, Seid M, Skarr D (2003). The PedsQL 4.0 as a pediatric population health measure: feasibility, reliability, and validity. Ambulatory Pediatrics: the Official Journal of the Ambulatory Pediatric Association.

[CR36] Varni JW, Seid M, Kurtin PS (2001). PedsQL 4.0: reliability and validity of the pediatric quality of life inventory version 4.0 generic core scales in healthy and patient populations. Medical Care.

[CR37] Varni JW, Seid M, Smith Knight T, Burwinkle T, Brown J, Szer IS (2002). The PedsQL in pediatric rheumatology: Reliability, validity, and responsiveness of the pediatric quality of life inventory generic core scales and rheumatology module. Arthritis and Rheumatism.

[CR38] Wright FV, Law M, Crombie V, Goldsmith CH, Dent P (1994). Development of a self-report functional status index for juvenile rheumatoid arthritis. The Journal of Rheumatology.

