# Supplementary material for: Health Related Quality of Life in Juvenile-Onset Systemic Lupus Erythematosus: A Questionnaire-Based Study
Source: Matern Child Health J. 2023 Jun 12;27(9):1578–88. doi: 10.1007/s10995-023-03680-x (PMC10359351; doi:10.1007/s10995-023-03680-x)
Supplement: Supplementary file 1 — Supplementary file1 (DOCX 41 KB) [file 10995_2023_3680_MOESM1_ESM.docx]

**Supplementary Table 1:** The main characteristics and findings of studies on HRQOL in children with SLE**.**

| **First author/ reference** | **Country** | **Number** | **Age /years** | **Tool used** | **Main findings** |
| --- | --- | --- | --- | --- | --- |
| **Eid et al /[current]** | Egypt | 100 | 8-18 | PedsQL^TM^4.0  PedsQL^TM^ 3 RM  SMILEY | The longer duration of illness, high cumulative steroid doses, higher SLEDAI and SDI scores, presence of obesity and low bone density were associated with low scores for all 3 tools. |
| **Putera et al. 2020** | Indonesia | 62  LN | 6-18 | PedsQL^TM^ 3 RM | Mental health disorders reduce the QoL of LN children, and the level of QoL in induction phase is lower than maintenance phase. |
| **Stevens et al. 2019** | USA | 42 | 9.9  (All prepubertal) | PedsQL^TM^4.0  PedsQL^TM^ 3 RM | *School functioning had the worst QOL measure  *Disease damage was associated with greater impact on Worry domain. |
| **Moorthy et al. 2017** | heterogenous ethnicity | 456 | 14±3 | SMILEY  PedsQL^TM^4.0  CHAQ | Female gender, high disease activity  and damage, and use of cyclophosphamide  and/rituximab are related to lower HRQOL scores. |
| **Rogers et al. 2017** | USA  (Heterogenous ethnicity) | 48 | 13.81±2.45 | PedsQL^TM^ 4.0. | School domain most affected, social domain least affected |
| **Levy et al. 2014** | Canada  Multiple ethnicities | 196 | 14.9±3 | CHQ | *SLE patients rated their HRQOL significantly more poorly in 9 of 10 individual domains, and in 4 of 10 domains when compared to a cohort of JIA |
| **Jones et al. 2013** | USA | 86 | 15.7±2.44 | PROMIS  CHQ | cSLE patients had markedly decreased HRQOL. None of the QoL measures correlated with the SLEDAI or MD-rated disease activity. |
| **Moorthy et al. 2013** | Brazil | 93 | 5-18 | PedsQL^TM^ 4.0  PedsQL 3.0 RM  CHAQ  SMILEY | SMILEY was found to have good psychometric properties (validity  and reliability). |
| **Moorthy et al. 2009** | USA (heterogenous ethnicity) | 68 | 7-18 | SMILEY | Changes in disease activity and damage measures correlated most strongly with the changes in SMILEY© domains, Limitation and Burden of SLE. |
| **Brunner et al. 2009** | North American | 98 | 14.7±0.5 | PedsQL^TM^ 4.0  PedsQL 3.0 RM  CHQ | HRQOL with cSLE is significantly lower than healthy populations. Higher disease activity and damage are associated with significantly lower HRQOL |
| **Houghton et al. 2008** | Canada | 15 | 12-19 years | K-FSS  CHQ | No significant correlation of fatigue with fitness measures. Neither fatigue nor fitness was significantly correlated with disease activity, disease damage, or QoL measures. |
| **Ruperto et al. 2004** | Multiple ethnicities | 297 | 16.2±4.9 | CHQ | The most impaired CHQ subscales were global health, general health  perceptions, and parent impact– emotional. The SLEDAI score was signiﬁcantly correlated with both the physical  summary score (r ⴝⴚ0.29, P < 0.0001) and psychosocial summary score (r ⴝⴚ0.25, P < 0.0001), whereas the SDI score  was signiﬁcantly correlated only with the physical summary score  The most impaired CHQ subscales were global health, general health  perceptions, and parent impact– emotional. The SLEDAI score was signiﬁcantly correlated with both the physical  summary score (r ⴝⴚ0.29, P < 0.0001) and psychosocial summary score (r ⴝⴚ0.25, P < 0.0001), whereas the SDI score  was signiﬁcantly correlated only with the physical summary score  The SLEDAI score was significantly correlated with physical and psychosocial summary scores, whereas the SDI score was significantly correlated only with the physical summary score |
| **Varni et al. 2002** | USA (heterogenous ethinicity) | 22 | 11.47±4.27 | PedsQL^TM^ 4.0.  PedsQL 3.0 RM | *PedsQL can differentiate between healthy and children with rheumatology disorders as a group. Total score in SLE patients was significantly lower than healthy children. |

PedsQL^TM^ 4: Pediatric Quality of Life Inventory, PedsQLTM 3.0 RM: the PedsQLTM 3.0 Rheumatology Module, SLE: systemic lupus erythematosus, SD: standard deviation, SDI: systemic lupus damage index. CGCs: cumulative glucocorticoids, SLEDAI: SLE disease activity index, CHQ: Child health questionnaire, PROMIS: Patient-Reported Outcomes Measurement Information System, LN: lupus nephritis, K-FSS:

[Kurtzke Functional Systems Scores.](https://www.yumpu.com/en/document/view/53529553/kurtzke-functional-systems-scores-kfss)
